# Electrophysiological analysis of cardiac K_ATP _channel

**DOI:** 10.52601/bpr.2024.240023

**Published:** 2025-04-30

**Authors:** Jianyi Huo, Hua-Qian Yang

**Affiliations:** 1 Cyrus Tang Medical Institute, Soochow University, Suzhou 215123, Jiangsu, China

**Keywords:** K_ATP_ channel, Cardiomyocyte isolation, Langendorff perfusion, Patch clamp

## Abstract

ATP-sensitive potassium (K_ATP_) channels are integral components in excitable cells, particularly in cardiomyocytes, serving as critical regulators of cellular metabolism and electrical excitability. In instances of prolonged oxygen deprivation or heightened metabolic requirements, the opening of K_ATP_ channels enables potassium efflux by virtue of a diminished ATP/ADP ratio. This process aids in maintaining membrane potential stability, thereby mitigating excessive excitability and cellular contraction, ultimately contributing significantly to cardiac protection. The accurate isolation of intact single cardiomyocytes and the electrophysiological evaluation of K_ATP_ channels are pivotal processes in research on K_ATP_ channels in cardiomyocytes *in vitro*. Here, we present a comprehensive protocol not only for the efficient isolation of viable cardiomyocytes from the adult mouse through the Langendorff perfusion method, but also for the recording of K_ATP_ channel currents in single cardiomyocytes employing patch clamp technique.

## INTRODUCTION

Cardiomyocytes, the contractile cells of the heart, have a specialized structure tailored for electrical signaling and efficient contraction. They contain organized sarcomeres, the basic contractile units composed of actin and myosin filaments, which facilitate muscle contraction (Risi *et al.* 2023; Wang and Raunser 2023). Cardiomyocytes are characterized by the presence of transverse tubules, which are deep invaginations of the cell membrane promoting rapid transmission of action potentials into the interior of the cell (Hong and Shaw 2017) to trigger synchronized calcium release from the sarcoplasmic reticulum. Additionally, cardiomyocytes are connected by intercalated discs, which contain gap junctions for electrical coupling and desmosomes for mechanical stability, ensuring coordinated and robust heartbeats (Franke *et al.* 2006; Patel and Green 2014).

Understanding the electrical signaling in cardiomyocytes is crucial for elucidating heart function. One of the most effective techniques for investigating these electrical properties is the patch clamp. The patch clamp technique, developed in the late 1970s and early 1980s by Erwin Neher and Bert Sakmann, revolutionized the field of electrophysiology (Hamill *et al.* 1981; Lux *et al.* 1981; Neher *et al.* 1978; Sakmann and Neher 1984; Sigworth and Neher 1980). This method allows for the recording of ionic currents through individual ion channels in the cell membrane. The initial procedure is to build a high-resistance seal with the cell membrane by use of a glass micropipette with a very fine tip. Once the seal is established, the membrane patch enclosed by the pipette tip can be excised (detached from the cell) or left attached to the cell, allowing for the study of ion channels in various configurations such as cell-attached, inside-out, outside-out or whole-cell mode (Hill and Stephens 2021).

In the whole-cell mode, the membrane under the patch pipette is ruptured, allowing electrical access to the entire cell, thus recording the ionic currents across the whole cell membrane. In the cell-attached mode, the patch remains attached to the cell, enabling the study of single channel activity within its natural cellular context. The inside-out and outside-out configurations are used to study ion channels regulated by intracellular and extracellular stimuli, respectively. This versatile technique provides detailed insights into the function and regulation of ion channels in cardiomyocytes, which are critical for maintaining normal heart rhythm and function (Jiang *et al.* 2022; Jurkat-Rott and Lehmann-Horn 2004; Kang *et al.* 2017; Ossola *et al.* 2015). The integration of patch clamp methods with studies on cardiomyocytes has significantly advanced our understanding of cardiac physiology and pathology, particularly in the context of ion channels like the ATP-sensitive potassium (K_ATP_) channels (Huang *et al.* 2018; Lv *et al.* 2022; Tuncay *et al.* 2023; Yang *et al.* 2020).

The K_ATP_ channels, first identified in cardiac cells in the early 1980s, are pivotal in linking intracellular metabolic status to membrane excitability across a variety of tissues (Noma 1983). K_ATP_ channels are especially significant in cardiac tissue, where they play crucial roles in modulating heart function in response to metabolic stress and ischemic conditions (Velez *et al.* 2022; Yang *et al.* 2016; Youssef *et al.* 2017).

K_ATP_ channel is a hetero-octamer consisting of four pore-forming subunits (Kir6.1 or Kir6.2) and four regulatory sulphonylureas receptor subunits (SUR1, SUR2A or SUR2B). Under normal conditions, high ATP levels keep the channels closed. While under metabolic stress, decreased intracellular ATP and increased ADP concentrations lead to channel opening. K_ATP_ channel opening allows potassium ions to flow out of the cell, leading to membrane hyperpolarization which reduces cellular excitability and energy consumption by decreasing calcium influx and myocardial contractility (Foster and Coetzee 2016).

The protective effects of K_ATP_ channels are also profound during episodes of ischemia. Activation of these channels during ischemic conditions has been shown as a precondition of the heart, enhancing its tolerance to subsequent ischemic stress through a mechanism known as "ischemic preconditioning", which is crucial for survival in acute cardiac events (Yang *et al.* 2016). Studies have demonstrated that pharmacological activation or genetic upregulation of K_ATP_ channels can significantly reduce infarct size and improve cardiomyocyte survival during myocardial infarction (Kobara *et al.* 2023; Pertiwi *et al.* 2019).

Recent advances in molecular biology and genetic engineering have provided new insights into the regulation of these channels. For instance, studies have explored how specific mutations within the Kir6.x or SURx subunits alter channel function, leading to changes in cardiac phenotypes (McClenaghan and Nichols 2022; Zhao *et al.* 2020). Additionally, emerging research suggests that K_ATP_ channel activity might be modulated not only by nucleotides but also by lipid-derived molecules and other signaling pathways, further broadening our reorganization of their physiological impacts (Driggers and Shyng 2023).

Several protocols for isolating cardiomyocytes have been published, while to the best of our knowledge, only one paper for the analysis of K_ATP_ channel electrophysiology has been documented to date (Aziz *et al*. 2020). The current protocol aims to provide an updated standard for the isolation of adult mouse cardiomyocytes and the precise recording of K_ATP_ channel current, essential for deciphering its role in cardiac physiology and pathology (Foster and Coetzee 2016; Tinker *et* *al.* 2018).

## APPLICATIONS AND ADVANTAGES OF THE PROTOCOL

This protocol allows for the precise isolation of cardiomyocytes and electrophysiological assessment of K_ATP_ channels, providing insights into their roles in cardiac physiology and pathology. The Langendorff perfusion method ensures high-quality and viable cardiomyocytes, essential for accurate electrophysiological recordings. The patch clamp technique was viewed as a gold standard method in the functional investigation of ion channels. Using inside-out and whole-cell patch clamp techniques, researchers can thoroughly examine their activity in both the intrinsic properties of K_ATP_ channels and their function in the intact cellular environment, which made the two methods more widely used. This protocol combines cardiomyocyte isolation with patch-clamp analysis to provide a comprehensive framework for researching K_ATP_ channels. By ensuring the reproducibility and reliability of experiments, it enhances our understanding of K_ATP_ channel functions over time. The protocol also facilitates the identification of potential therapeutic targets for cardiac diseases and supports advancements in cardiovascular research.

## LIMITATIONS OF THE PROTOCOL

The protocol for analyzing K_ATP_ channel function in mouse cardiomyocytes has several limitations. First, the Langendorff perfusion method requires precise technical skills and equipment, making it challenging for less experienced researchers and those with limited resources. Second, variability in cell yield and quality can result from minor deviations during the isolation process. The protocol's reliance on specific reagents like Type II collagenase necessitates careful control, as both under- and over-digestion can compromise cell viability. Moreover, electrophysiological recordings are susceptible to inconsistencies due to variations in electrode handling and cell selection. Specialized equipment such as patch clamp amplifiers and vibration isolation tables are essential but may not be available in all laboratories. Last but not the least, the labor-intensive and time-consuming nature of the protocol also limits its suitability for high-throughput studies or researchers with limited electrophysiological experience.

## OVERVIEW OF THE PROTOCOL

**Figure 1:**
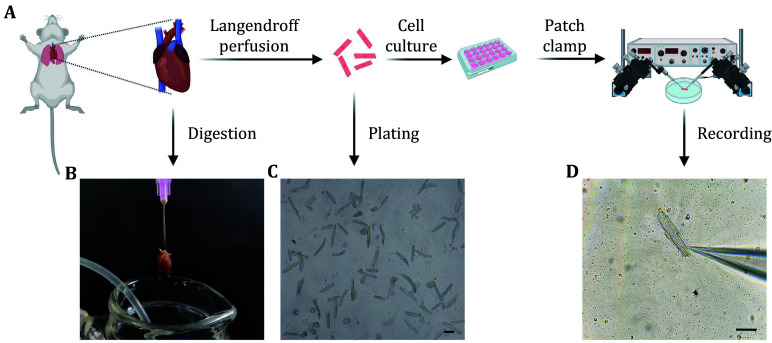
Workflow of mouse cardiomyocyte isolation and patch clamp recording. **A** Langendorff perfusion system. Schematic representation of the Langendorff method for isolating cardiomyocytes. The heart is excised from the mouse, followed by perfusion with an enzyme-containing solution to dissociate cells. **B** Heart digestion. Image of the mouse heart being digested using the Langendorff perfusion system. The perfusion needle is inserted into the aorta, and the heart is perfused with collagenase solution. **C** Isolated cardiomyocytes. Light microscopy image showing isolated adult mouse cardiomyocytes. The cells are shown to disperse after enzymatic digestion. Scale bar, 50 μm. **D** Patch clamp setup. Image of a cardiomyocyte being patched with a glass micropipette. The cell is positioned under an inverted microscope, and the pipette is used to form a high-resistance seal for recording. Scale bar, 50 μm

This protocol outlines, for the first time, both the isolation of mouse cardiomyocytes and electrophysiological assessment of K_ATP_ channels (Fig. 1). Using the Langendorff method, mouse hearts are perfused with calcium-free Tyrode's Buffer and Type II collagenase to release viable cardiomyocytes. The isolated cells are seeded on laminin-coated coverslips for stability. Electrophysiological analysis employs two patch clamp configurations: inside-out and whole-cell modes. The inside-out patch clamp method focuses on the intrinsic properties of K_ATP_ channels by isolating a membrane patch and exposing it to various solutions. The whole-cell patch clamp technique assesses channel activity within the intact cellular environment, providing insights into their physiological roles. The protocol includes detailed steps for solution preparation, electrode handling and patch clamp execution.

## MATERIALS AND EQUIPMENT

### Materials

• Sodium heparin (≥140USP units/mg) (Solarbio, Cat. No. H8060)

• Isoflurane (RWD, Cat. No. R510-22-10)

• NaCl (Sigma, Cat. No. 71376)

• KCl (Sigma, Cat. No. P3911)

• HEPES (Sigma, Cat. No. H4034)

• MgCl_2_ (1 mol/L in H_2_O) (Sigma, Cat. No. 63069)

• NaH_2_PO_4_ (SCR, Cat. No. 2004081933)

• CaCl_2_ (1 mol/L in H_2_O) (Sigma, Cat. No. 21115)

• Glucose (Sigma, Cat. No. G7528)

• Type II Collagenase (Worthington, Cat. No. LS004176)

• Taurine (Macklin, Cat. No. T818825)

• L-Glutamic Acid (Macklin, Cat. No. L810369)

• EGTA (Sigma, Cat. No. E3889)

• KH_2_PO_4_ (SCR, Cat. No. 10017608)

• Penicillin-Streptomycin (Macklin, Q6532)

• MEM Medium (Procell, Cat. No. PM150410)

• Laminin (Sigma, Cat. No. L2020)

• Potassium Gluconate (Sigma, Cat. No. G4500)

• MgATP (Sigma, Cat. No. A9187)

• DMSO (Absin, Cat. No. abs9187)

• Pinacidil (Sigma, Cat. No. P154)

• Glibenclamide (Sigma, Cat. No. G0639)

• Glass Coverslips (Solarbio, Cat. No. YA0782)

• Normal saline (Solarbio, Cat. No. IN9000)

### Equipment

• Analog-to-Digital Converter (AXON, Digidata1550B)

• Amplifier (AXON, Axopatch200B Amplifier)

• Pipette Puller (Sutter Instrument, Model P97)

• Micromanipulator (Sutter Instrument, MP-285)

• Langendorff Perfusion System

• Perfusion system (MappingLab, PVG-08)

• Vibration Isolation Table (LSXPT, 12-075)

• Inverted fluorescent microscope (Nexcope, NIB610FL)

• pH Meter (Mettler ToleDO, FE28-standard)

## EXPERIMENTAL PROCEDURES

### Isolation of adult mouse cardiomyocytes

#### Preparation of Tyrode’s buffer [TIMING ~30 min]

Dissolve the components (see Table 1) in deionized water and make up the volume to 500 mL. Adjust the pH to 7.35–7.4 with sodium hydroxide (NaOH). Store at 4 °C and use up within one week. For calcium-containing Tyrode’s Buffer, add calcium chloride (CaCl_2_) to a final concentration of 1.8 mmol/L.

**Table 1:** Ingredients used for preparing Tyrode’s buffer

Ingredients	Final concentration	Amount
Sodium chloride (NaCl)	137 mmol/L	4.003 g
Potassium chloride (KCl)	5.4 mmol/L	0.201 g
Sodium dihydrogen phosphate (NaH_2_PO_4_)	0.33 mmol/L	0.020 g
4-(2-Hydroxyethyl)piperazine-1-ethanesulfonic acid (HEPES)	10 mmol/L	1.192 g
Glucose	10 mmol/L	0.900 g
Taurine	10 mmol/L	0.621 g
Magnesium chloride (MgCl_2_)	1 mmol/L	0.5 mL

**[CRITICAL** **STEP]** Ensure the pH adjustment is precise to maintain proper buffer function. The initial pH of the Tyrode’s solution is usually between 5 and 6, and it should be adjusted to 7.35–7.4 with NaOH. Also, ensure the solution is at the experimental temperature when adjusting the pH to prevent discrepancies due to temperature-related pH shifts.

#### Preparation of Kraft-Bruhe (KB) solution [TIMING ~45 min]

Dissolve the components (see Table 2) in deionized water. EGTA is difficult to dissolve, so add it first and adjust the pH to around 10 with potassium hydroxide (KOH). Once EGTA is fully dissolved, add the remaining components. Make up the volume to 1000 mL, adjust the pH to 7.2 using KOH, and store at 4 °C and use up within one week.

**Table 2:** Ingredients used for preparing Kraft-Bruhe (KB) solution

Ingredients	Final concentration	Amount
Taurine	20 mmol/L	2.48 g
Glutamic Acid	50 mmol/L	7.36 g
4-(2-Hydroxyethyl)piperazine-1-ethanesulfonic acid (HEPES)	10 mmol/L	2.38 g
Glucose	10 mmol/L	1.80 g
Ethylene Glycol Tetraacetic Acid (EGTA)	1 mmol/L	0.38 g
Potassium Dihydrogen Phosphate (KH_2_PO_4_)	20 mmol/L	2.72 g
Potassium Chloride (KCl)	20 mmol/L	2.98 g
Magnesium Chloride (MgCl_2_)	3 mmol/L	3 mL

**[CRITICAL** **STEP]** Thoroughly dissolve EGTA before adding other components to ensure proper solution preparation.

#### Preparation of Type II collagenase solution [TIMING ~20 min]

Dissolve 31.2 mg Type II collagenase in 20 mL calcium-free Tyrode’s buffer and preheat to 37 °C. Prepare freshly and use immediately; it is not recommended for storage and should be used within the day of preparation.

#### Laminin coating [TIMING ~1 h]

Apply laminin (20 μg/mL, L2020, Sigma) evenly on sterilized glass coverslips and air dry at room temperature under sterile conditions. Prepare freshly before use to avoid contamination and use immediately.

**[CRITICAL** **STEP]** Ensure uniform laminin coating to promote consistent cell attachment.

#### Preparation of sodium heparin solution [TIMING ~15 min]

Weigh 70 mg of heparin sodium powder (≥140USP units/mg), dissolve it in 10 mL of normal saline, and stir thoroughly. Store the solution in a light-protected container at 4 °C and use up within one week.

#### Cleaning the Langendorff perfusion system [TIMING ~30 min]

Wash the Langendorff perfusion system twice with 75% ethanol, sterile water, and calcium-free Tyrode’s Buffer. Fill with prepared Type II collagenase solution, ensuring the outflow temperature at the perfusion needle is maintained at 37 °C.

#### Mouse heart perfusion [TIMING ~45 min]

Adult mouse (9–11 weeks old), obtained from Vital River, is intraperitoneally injected 200 µL heparin sodium solution. After ten minutes to ensure proper anticoagulation, the mouse is anesthetized with isoflurane. Dissect out the heart and place it in pre-chilled calcium-containing Tyrode’s Buffer (to promote contraction and pump out blood). Trim excess tissues and locate the aorta. Fit the aorta onto a needle covered with parafilm and secure it with surgical thread. Perfuse with calcium-free Tyrode’s Buffer to flush out residual blood, then switch to 1.56 mg/mL Type II collagenase solution (preheated to 37 °C) for 12 min. Gently squeeze the heart with tweezers to check the digestion status. The heart becomes soft if adequately digested.

**[CRITICAL** **STEP]** Monitor digestion closely to avoid over-digestion, which can damage cardiomyocytes. The criteria for stopping digestion include several key observations to ensure optimal cell viability. (1) Softening of the heart tissue. The heart should become soft and pliable, indicating that the extracellular matrix has been sufficiently digested without damaging the cells. (2) Cell yield and quality. Collect a drop of perfused solution and observe under a microscope. The drop should contain 5–10 rod-shaped, striated cardiomyocytes. (3) Time monitoring. Typically, digestion should not exceed 15 min to avoid over-digestion.

#### Collecting cardiomyocytes [TIMING ~35 min]

Remove the digested heart, mince it finely with surgical scissors, and add KB solution. Wait for 10 s to allow the tissue fragments to sink, then transfer the supernatant to a 50 mL centrifuge tube. Repeat several times to increase cell yield. Leave the collected cardiomyocytes for 10 min to sink down, remove the supernatant, resuspend in KB solution, and repeat this wash step twice.

#### Seeding cardiomyocytes for attachment [TIMING ~60 min]

Replace the KB solution with a pre-warmed MEM complete medium (99% MEM medium, 1% penicillin-streptomycin). Resuspend and incubate the cells in the MEM complete medium. Count the cells using a hemocytometer, and seed 2–4 × 10⁴ cells onto laminin-coated round coverslips in 24-well plates. Incubate at 37 °C for 1 h to allow cells to attach, as this temperature mimics physiological conditions and provides an optimal environment for cell viability and adhesion. Maintain a humidified atmosphere with 5% CO_2_ to ensure proper pH balance and cellular environment. Allow the cells to attach for approximately 1 h before proceeding with further experiments or medium changes.

**[CRITICAL** **STEP]** Ensure accurate cell counting and gentle seeding to promote proper cell attachment.

#### Changing medium for cardiomyocytes [TIMING ~20 min]

Remove the supernatant and add MEM complete medium to wash away non-adherent and dead cells. Proceed with subsequent experiments.

### Inside-out patch clamp of mouse cardiomyocytes

#### Reagent preparation

##### Inside-out pipette solution [TIMING ~30 min]

Dissolve the components (see Table 3) in deionized water, make up to 50 mL, and adjust the pH to 7.4 with KOH. Aliquot 1 mL into 1.5 mL Eppendorf tubes, and store at 4 °C for up to one week, while aliquots can be stored at –20 °C for up to one month.

**Table 3:** Ingredients used for preparing inside-out pipette solution

Ingredients	Final concentration	Amount
Potassium chloride (KCl)	30 mmol/L	0.112 g
Calcium chloride (CaCl_2_)	2 mmol/L	50 μL
Magnesium chloride (MgCl_2_)	1 mmol/L	50 μL
4-(2-Hydroxyethyl)piperazine-1-ethanesulfonic acid (HEPES)	10 mmol/L	0.119 g
Potassium gluconate	110 mmol/L	1.288 g

**[CRITICAL** **STEP]** Adjust the pH carefully to ensure proper solution functionality.

##### Inside-out base solution [TIMING ~30 min]

Dissolve the components (see Table 4) in deionized water, make up to 500 mL, adjust the pH to 7.35–7.4 with KOH, and store at 4 °C and use up within one week.

**Table 4:** Ingredients used for preparing inside-out base solution

Ingredients	Final Concentration	Amount
Potassium Chloride (KCl)	30 mmol/L	0.112 g
Ethylene Glycol Tetraacetic Acid (EGTA)	1 mmol/L	0.190 g
4-(2-Hydroxyethyl)piperazine-1-ethanesulfonic acid (HEPES)	10 mmol/L	0.119 g
Magnesium Chloride (MgCl_2_)	1 mmol/L	500 μL
Potassium Gluconate	110 mmol/L	1.288 g

#### Equipment setup [TIMING ~35 min]

Power on the main power supply and the stabilizer, turn on the computer, amplifier, and analog-to-digital converter, and start the Clampex software. Pull several glass pipettes with the puller and check the vibration isolation table. Turn on the inverted microscope and micromanipulator. Prepare 1 mmol/L MgATP in K_ATP_ external solution and load it into different perfusion tubes.

#### Glass pipette pulling and resistance measurement [TIMING ~20 min]

With our current RAMP temperature of 598 °C, the puller program is set as follows:

(1) HEAT = 600, PULL = 0, VEL = 27, TIME = 240; (2) HEAT = 580, PULL = 0, VEL = 28, TIME = 240; (3) HEAT = 560, PULL = 0, VEL = 28, TIME = 240; (4) HEAT = 550, PULL = 0, VEL = 28, TIME = 240.

Fill the glass pipette with pipette solution, remove air bubbles and place the glass pipette in the pipette holder and tighten the knob. Apply a slight positive pressure to avoid tip block and immerse it in the external solution. Ensure the electrode resistance is around 4–5 MΩ.

**[CRITICAL** **STEP]** Ensure pipette resistance is within the acceptable range to gain signal quality.

#### Select cells [TIMING ~8 min]

Put the coverslip with cardiomyocytes in a 60 mm dish with an external solution. Under the inverted microscope at low magnification, select healthy cells by shape and confirm their status under high magnification, and the cells with a rectangle shape and clear striated pattern are recognized as healthy cells. Adjust the micromanipulator to position the pipette tip on top of the selected cell. Adjust the liquid junction to ensure the baseline is at zero.

**[CRITICAL** **STEP]** Select only healthy cells for accurate and reproducible results.

#### Seal formation [TIMING ~10 min]

**Figure 2:**
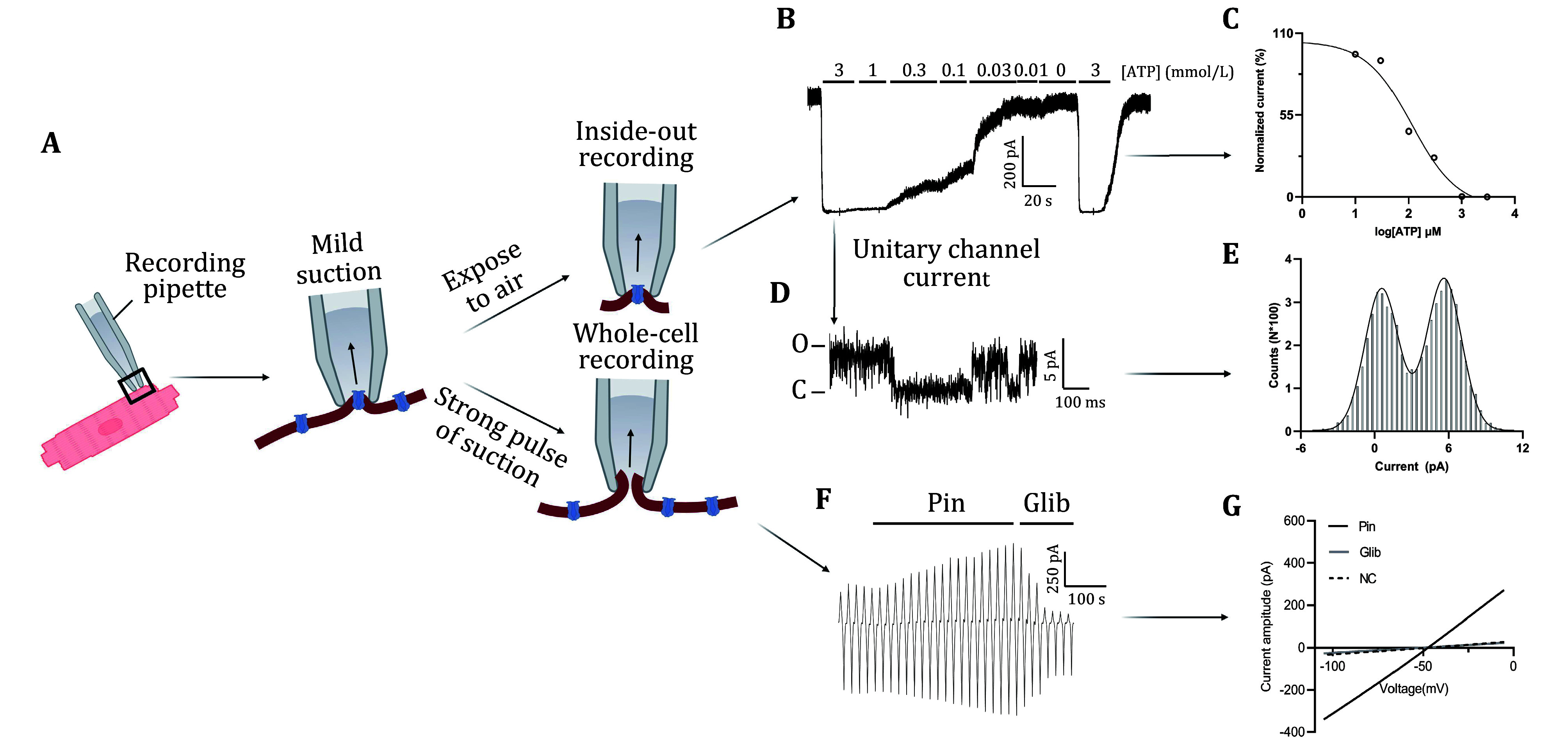
Patch clamp recording and data analysis of K_ATP_ channel currents. **A** Patch-clamp methodology. Schematic representation of the patch-clamp technique used to record K_ATP_ channel currents. The process begins with the placement of the recording pipette on a cardiomyocyte, followed by mild suction to form a giga-seal. Two configurations are shown: inside-out and whole-cell modes. The inside-out patch involves exposing the intracellular side of the channel to the extracellular environment, whereas the whole-cell patch configuration allows for the recording of currents from the entire cell. **B** Inside-out current recording. Representative trace of K_ATP_ channel currents recorded in the inside-out patch configuration in response to varying ATP concentrations. The ATP concentrations tested (in mmol/L) are indicated above the trace. **C** ATP sensitivity curve: Normalized current as a function of ATP concentration. The current is plotted on a logarithmic scale, demonstrating the inhibitory effect of ATP on K_ATP_ channel activity. **D** Single-channel current trace. High-resolution current trace showing unitary K_ATP_ channel openings and closings, recorded under conditions that allow single channel activity. The annotations on the trace indicate that "O" (Open) represents the channel's open state, and "C" (Close) represents the channel's closed state. **E** Current amplitude histogram. Distribution of single-channel current amplitudes, illustrating the presence of distinct conductance states. **F** Whole-cell current recording. Current trace showing K_ATP_ channel activity in whole-cell configuration. The trace includes the effects of pinacidil (Pin, a K_ATP_ channel opener) and glibenclamide (Glib, a K_ATP_ channel blocker). **G** Current–voltage relationship. Current–voltage (*I*–*V*) curve demonstrating the effect of pinacidil and glibenclamide on K_ATP_ channel currents. The *I*–*V* relationship is shown for control (NC), pinacidil-treated (Pin), and glibenclamide-treated (Glib) conditions

Lower the micromanipulator speed and gently move the pipette tip close to the cell. When the baseline square wave suddenly decreases, stop lowering the pipette and apply negative pressure via the syringe to form a seal. A successful seal is indicated by a quick decrease in the square wave and an increase in resistance to GΩ. Compensate the fast capacitance current. Once stable, move the electrode tip away from the cell, tearing a small patch of the cell membrane, and forming an inside-out patch (Fig. 2).

#### Perfusion and current recording [TIMING ~20 min]

Move the electrode tip to the perfusion device outlet. At +80 mV, switch K_ATP_ external solutions with or without 1 mmol/L ATP to record K_ATP_ current (Fig. 2).

### Whole-cell patch clamp of mouse cardiomyocytes

#### Reagent preparation

##### Whole-cell pipette solution [TIMING ~30 min]

Dissolve the components (see Table 5) in deionized water, make up to 50 mL, adjust the pH to 7.2 with KOH. Aliquot 1 mL into 1.5 mL Eppendorf tubes, and store at 4 °C for up to one week, while aliquots can be stored at –20 °C for up to one month.

**Table 5:** Ingredients used for preparing whole-cell pipette solution

Ingredients	Final concentration	Amount
Potassium chloride (KCl)	140 mmol/L	0.522 g
Sodium chloride (NaCl)	10 mmol/L	0.029 g
Ethylene glycol tetraacetic acid (EGTA)	1 mmol/L	0.019 g
4-(2-Hydroxyethyl)piperazine-1-ethanesulfonic acid (HEPES)	10 mmol/L	0.119 g
MgATP	1 mmol/L	0.025 g

##### Whole-cell base solution [TIMING ~30 min]

Dissolve the components (see Table 6) in deionized water, make up to 500 mL, adjust the pH to 7.35–7.4 with NaOH, and store at 4 °C and use up within one week.

**Table 6:** Ingredients used for preparing whole-cell base solution

Ingredients	Final concentration	Amount
Potassium chloride (KCl)	5.4 mmol/L	0.201 g
Sodium chloride (NaCl)	137 mmol/L	4.003 g
Magnesium chloride (MgCl_2_)	1 mmol/L	500 μL
4-(2-Hydroxyethyl)piperazine-1-ethanesulfonic acid (HEPES)	10 mmol/L	1.192 g
Glucose	10 mmol/L	0.901 g
Calcium chloride (CaCl_2_)	2 mmol/L	1 mL

Preparation of pinacidil and glibenclamide solutions

Both pinacidil and glibenclamide should be prepared freshly before the experiment. Pinacidil is dissolved in DMSO to create a 100 mmol/L stock solution, which is then diluted in a whole-cell bath solution to a final working concentration of 100 μmol/L. Similarly, glibenclamide is prepared as a 10 mmol/L stock solution in DMSO and diluted to a final working concentration of 5 μmol/L in the bath solution.

#### Equipment setup and cell selection

Same as inside-out patch clamp steps.

#### Seal formation and membrane rupture [TIMING ~10 min]

After the GΩ seal is achieved, wait for 3 min to let the seal stable. At a holding potential of –55 mV, with leak current <20 pA, apply significant negative pressure for membrane rupture (Fig. 2). Observe baseline fluctuations during rupture. After rupture, two large slow capacitance currents show up before and after the baseline. Check the leak current value; if it exceeds 50 pA, the cell is unqualified for further experiments. If the leak current is normal (<20 pA), compensate for slow and fast capacitance currents. Record membrane resistance (Rm, normal 500 MΩ–1 GΩ) and membrane capacitance values.

#### Perfusion and current recording [TIMING ~20 min]

Set the membrane potential at –55 mV and record currents with a ramp protocol (adjusting potential from –5 mV to –105 mV at a rate of –25 mV/s). Dissolve the K_ATP_ channel opener Pinacidil or blocker Glibenclamide in DMSO and dilute in external solution to final concentrations of 100 μmol/L and 5 μmol/L, respectively. Perfuse these solutions sequentially onto the cell and whole-cell K_ATP_ channel current is recognized as the difference between Pinacidil and Glibenclamide perfusion (Fig. 2).

### Data analysis

#### ATP sensitivity curve analysis

Figure 2C shows a dose-response curve obtained using the inside-out patch-clamp configuration, illustrating the relationship between ATP concentration and the normalized K_ATP_ channel current.

The curve in panel C represents the inhibitory effect of ATP on K_ATP_ channel activity. As ATP concentration increases, the K_ATP_ channel current decreases. This is quantified by plotting the normalized current (as a percentage of maximum activity) against the logarithm of ATP concentration. The curve is typically fitted with a sigmoidal function to determine the half-maximal inhibitory concentration (IC50) of ATP, which indicates the concentration of ATP required to reduce the channel activity by half. This analysis is crucial for understanding how ATP regulates K_ATP_ channel activity and provides insights into the channel's sensitivity to intracellular metabolic states.

#### Single-channel current analysis

Figure 2E displays a histogram of single-channel current amplitudes recorded using the inside-out patch-clamp configuration.

The histogram in panel E illustrates the distribution of current amplitudes observed in single-channel recordings of the K_ATP_ channel. The peaks in the histogram correspond to the most frequently observed conductance states, typically representing the open and closed states of the channel. By analyzing these peaks, researchers can deduce the conductance levels of the channel and the probability of the channel being open or closed at any given time. This information is vital for understanding the channel's gating behavior and how it transitions between different states.

#### Current–voltage (I–V) relationship

Figure 2G presents the current–voltage (*I*–*V*) relationship for K_ATP_ channel activity recorded under various conditions using the whole-cell patch-clamp configuration.

The *I*–*V* curve in panel G depicts the relationship between membrane potential and current flow through the K_ATP_ channels under different conditions, such as with and without pharmacological agents. This curve typically includes traces for control conditions (NC), as well as traces where the K_ATP_ channel is modulated by agents like pinacidil (a channel opener) and glibenclamide (a channel blocker). By examining the slope of the *I*–*V* curves, researchers can determine the conductance of the channels and how it changes in response to these agents. This analysis is essential for assessing the channel's functional properties and its response to therapeutic interventions.

## CONCLUSION

This protocol outlines a systematic approach for isolating mouse cardiomyocytes, followed by the electrophysiological analysis of K_ATP_ channels using the patch-clamp technique. The initial isolation step ensures the acquisition of viable cardiomyocytes by carefully controlling conditions to maintain cell integrity. Following isolation, the patch-clamp technique is employed to assess the functional properties of K_ATP_ channels within the cardiomyocytes. This process provides detailed insights into the channel's activity, its sensitivity to ATP, and its response to pharmacological agents. Such insights are crucial for understanding the role of K_ATP_ channels in cardiac physiology and pathology, and they contribute to the development of targeted therapies for cardiovascular diseases.

## Conflict of interest

Jianyi Huo and Hua-Qian Yang declare that they have no conflict of interest.
